# Identification of a novel risk factor for chronic wasting disease (CWD) in elk: S100G single nucleotide polymorphism (SNP) of the prion protein gene (*PRNP*)

**DOI:** 10.1186/s13567-023-01177-7

**Published:** 2023-06-16

**Authors:** Yu-Ran Lee, Yong-Chan Kim, Sae-Young Won, Min-Ju Jeong, Kyung-Je Park, Hoo-Chang Park, In-Soon Roh, Hae-Eun Kang, Hyun-Joo Sohn, Byung-Hoon Jeong

**Affiliations:** 1 Reference Laboratory for CWD, Foreign Animal Disease Division, Animal and Plant Quarantine Agency, Gimcheon, 39660 Republic of Korea; 2 Department of Biological Sciences, Andong National University, Andong, 36729 Republic of Korea; 3 Korea Zoonosis Research Institute, Jeonbuk National University, Iksan, 54531 Republic of Korea; 4 Department of Bioactive Material Sciences and Institute for Molecular Biology and Genetics, Jeonbuk National University, Jeonju, 54896 Republic of Korea

**Keywords:** CWD, prion, elk, S100G, *PRNP*, SNP, risk factor

## Abstract

Prion diseases are fatal and malignant infectious encephalopathies induced by the pathogenic form of prion protein (PrP^Sc^) originating from benign prion protein (PrP^C^). A previous study reported that the M132L single nucleotide polymorphism (SNP) of the prion protein gene (*PRNP*) is associated with susceptibility to chronic wasting disease (CWD) in elk. However, a recent meta-analysis integrated previous studies that did not find an association between the M132L SNP and susceptibility to CWD. Thus, there is controversy about the effect of M132L SNP on susceptibility to CWD. In the present study, we investigated novel risk factors for CWD in elk. We investigated genetic polymorphisms of the PRNP gene by amplicon sequencing and compared genotype, allele, and haplotype frequencies between CWD-positive and CWD-negative elk. In addition, we performed a linkage disequilibrium (LD) analysis by the Haploview version 4.2 program. Furthermore, we evaluated the 3D structure and electrostatic potential of elk prion protein (PrP) according to the S100G SNP using AlphaFold and the Swiss-PdbViewer 4.1 program. Finally, we analyzed the free energy change of elk PrP according to the S100G SNP using I-mutant 3.0 and CUPSAT. We identified 23 novel SNP of the elk* PRNP* gene in 248 elk. We found a strong association between* PRNP* SNP and susceptibility to CWD in elk. Among those SNP, S100G is the only non-synonymous SNP. We identified that S100G is predicted to change the electrostatic potential and free energy of elk PrP. To the best of our knowledge, this was the first report of a novel risk factor, the S100G SNP, for CWD.

## Introduction

Prion diseases are fatal and infectious neurodegenerative disorders caused by a highly aggregated and proteinase K-resistant form of prion protein (PrP^Sc^) converted from normal prion protein (PrP^C^) encoded by the prion protein gene (*PRNP*) [1–3]. In the Cervidae family, prion disease is called chronic wasting disease (CWD) and has been reported in various Cervidae species, including elk, mule deer, red deer, and sika deer [4–6]. Notably, although certain individuals have been infected with CWD, certain individuals have shown resistance to CWD on the farms where CWD occurred [7]. As the cause of this phenomenon, several studies have suggested that genetic polymorphisms of the* PRNP* gene play a pivotal role in susceptibility/resistance to CWD [8–10].

According to Monello et al., there was a correlation between the frequency of the 132L allele and CWD prevalence in 1018 elk sampled from various populations in the USA [11]. In addition, Haley et al. demonstrated that the 132MM genotype was nearly 2 to 3.5 times more prevalent in CWD-positive elk compared to the 132ML and 132LL genotypes, respectively [12]. White et al. also found that the 132L allele was less observed among CWD cases in 559 captive and free-ranging elk from a different geographic region in the USA [13]. However, other studies did not find that the genotype and allele frequencies of the M132L single nucleotide polymorphism (SNP) were associated with susceptibility to CWD in the USA and Korea [14, 15]. In addition, a meta-analysis of the three previous studies also did not identify a relationship between the M132L SNP and susceptibility to CWD in all genetic models [15]. Furthermore, real-time quaking-induced conversion (RT-QuIC) shows that the conversion efficiency of PrP^Sc^ of a specific genotype was not high but that the conversion efficiency of PrP^Sc^ was high when the genotype of the codon was identical between the template and seed [16]. These discrepancies may be linked to the sample size or CWD strains.

In Korea, more than 12 000 elk are bred, and recently, intermittent CWD cases have been reported there [17–19]. The exact cause of CWD is unknown since elk have been banned from importation from Canada since 2000. Since CWD is an extremely infectious disease, investigation of the novel risk factor for CWD is needed for preemptive control of CWD, a national disaster-type disease.

In the present study, to identify novel risk factors for CWD in elk, we investigated genetic polymorphisms of the* PRNP* gene and compared genotype, allele, and haplotype frequencies between 52 CWD-positive and 196 CWD-negative elk. In addition, we performed a linkage disequilibrium (LD) analysis among *PRNP* polymorphisms to find the LD relationship among *PRNP* polymorphisms. Furthermore, we analyzed the 3D structure and electrostatic potential of elk prion protein (PrP) according to the S100G SNP using AlphaFold and the Swiss-PdbViewer 4.1 program [20, 21]. Finally, we investigated the free energy change of elk PrP according to the S100G SNP using I-mutant 3.0 and CUPSAT [22, 23].

## Materials and methods

### Ethics statements

All experimental procedures were approved by the Institutional Animal Care and Use Committee of Jeonbuk National University (IACUC Number: JBNU-2019-0076). All experiments were carried out following the Korea Experimental Animal Protection Act.

### Subjects

Brain tissues derived from 248 elk were obtained from 6 animal farms in the Republic of Korea including Chungnam (Geumsan, 61 animals; Hongsung, 19 animals), Gyeongnam (Namhae, 50 animals; Jinju, 77 animals), and Jeonnam (Hampyeong, 2 animals; Gokseong, 39 animals) provinces where CWD has occurred [12]. The breeding scale of each farm is as follows, Chungnam (Geumsan), 61 animals; Gyeongnam (Namhae), 56 animals; Jeonnam (Gokseong), 53 animals; Jeonnam (Hampyeong); 221 animals. The breeding scale of Chungnam (Hongsung) and Gyeongnam (Jinju) was not available. The owners of the farms in Chungnam (Geumsan) and Gyeongnam (Namhae) were the same, however, the epidemiological association (route and source of infection) between each farm was not observed. CWD tests were conducted on all brain samples by the Animal and Plant Quarantine Agency (APQA) in the Republic of Korea using the HerdChek BSE-Scrapie Antigen Kit (IDEXX, USA) and Western blot analysis. Out of the 248 elk, 52 elk (Gyeongnam, 19 animals; Jeonnam, 19 animals (Gokseong, 17 animals; Hampyeong, 2 animals); Chungnam, 14 animals) were diagnosed with CWD.

### Genomic DNA

Genomic DNA was isolated from 20 mg of brain tissue using a QIAamp DNA Mini Kit (Qiagen, Hilden, Germany) following the manufacturer’s protocol.

### Genetic analysis of the elk *PRNP* gene

Polymerase chain reaction (PCR) was conducted to investigate the variations from amino acid 8 to 235 within the open reading frame (ORF) of elk *PRNP* gene (accession number: FJ590751.1) from the genomic DNA using the forward and reverse gene-specific primers PRNP-F (ATGGTGAAAAGCCACATAGGC) and PRNP-R (ACACTTGCCCCTCTTTGGTA). PCR was performed using DNA Polymerase (Biofact, Daejeon, Republic of Korea) and an S-1000 Thermal Cycler (Bio-Rad, Hercules, California, USA) according to the manufacturer’s protocol. The PCR conditions for the PRNP-F and PRNP-R primers were as follows: 95 °C for 2 min for denaturation; 35 cycles of 94 °C for 45 s, 59 °C for 45 s, and 72 °C for 1 min 30 s; and 1 cycle of 72 °C for 10 min for extension. Detailed information on PCR is described in a previous study [12]. The amplicons were eluted using a PCR Purification Kit (Thermo Fisher Scientific, Bridgewater, New Jersey, USA) and sequenced by an ABI 3730 automatic sequencer (ABI, Foster City, California, USA) on both strands. Sequencing results were visualized by Finch TV software (Geospiza Inc., Seattle, USA), and genotyping of each nucleotide (Q > 40) was performed.

### Statistical analysis

Statistical analyses were conducted by SAS version 9.4 (SAS Institute Inc., USA). The differences in genotype and allele distributions of the* PRNP* gene between CWD-negative and CWD-positive elk were analyzed using the χ^2^ test and Fisher exact test. The Hardy-Weinberg equilibrium (HWE), haplotype analyses and LD tests were conducted by Haploview version 4.2 (Broad Institute, Cambridge, MA, USA) as previously described [8].

### 3D structure and electrostatic potential analyses of elk PrP

The 3D structure of elk PrP was predicted by AlphaFold based on machine learning. The confidence of modeling was evaluated by the predicted local distance difference test (pLDDT) score on a scale from 0–100. The predicted structure was visualized by the Swiss-PdbViewer 4.1 program.

### Prediction of protein stability changes

Protein stability changes according to S100G were predicted by I-mutant 3.0 and CUPSAT. I-mutant 3.0 estimated protein stability changes based on a support vector machine (SVM) and evaluated the free energy change (DDG) value with positive (increase) and negative (decrease) signs. CUPSAT calculated protein stability changes based on protein environment-specific mean force potentials and evaluated the DDG value with positive (increase) and negative (decrease) signs.

## Results

### Identification of 23 novel* PRNP* polymorphisms in elk

**Figure 1:**
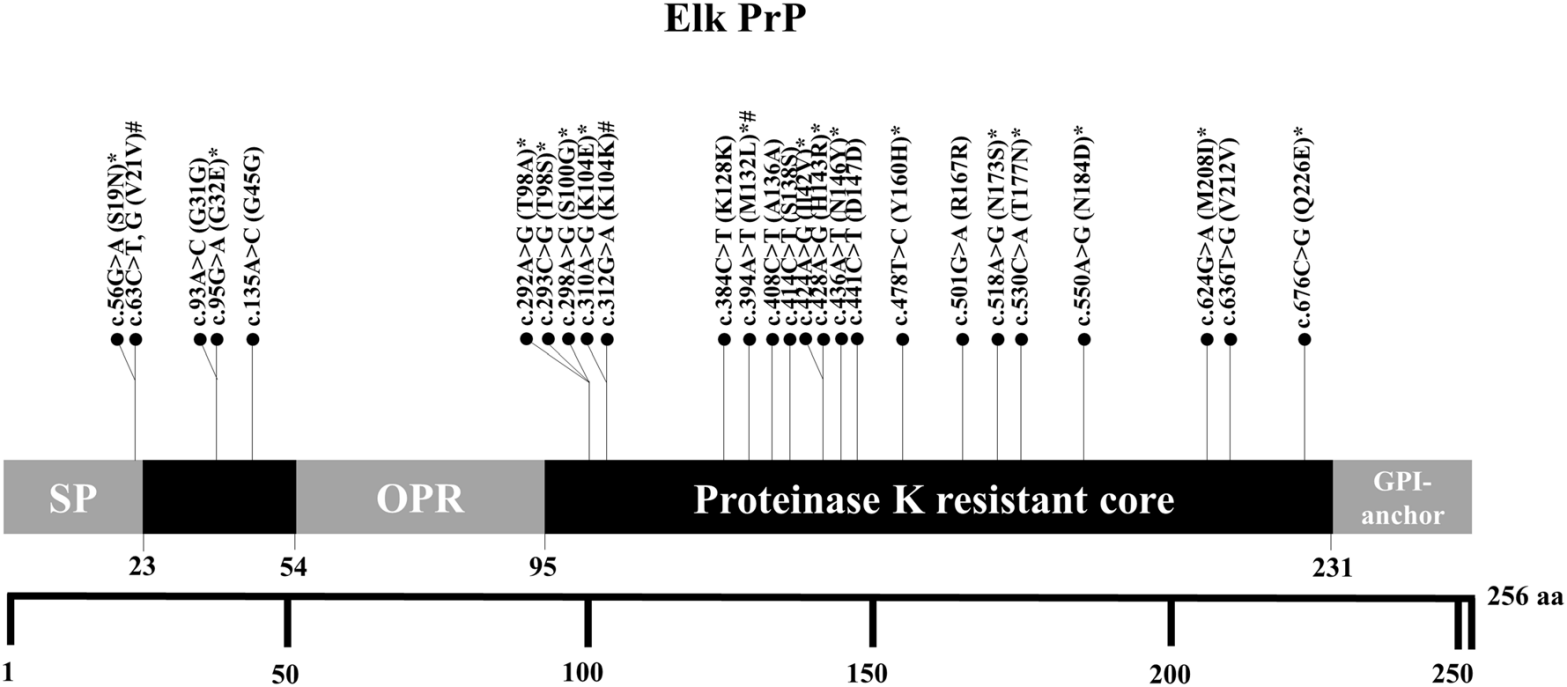
**Schematic map of the prion protein (PrP) with single-nucleotide polymorphisms (SNP) of the prion protein gene (*PRNP*) in elk.** * indicates non-synonymous SNP. # indicates previously reported non-synonymous SNP. SP: signal peptide; OPR: octapeptide repeat region.

**Figure 2:**
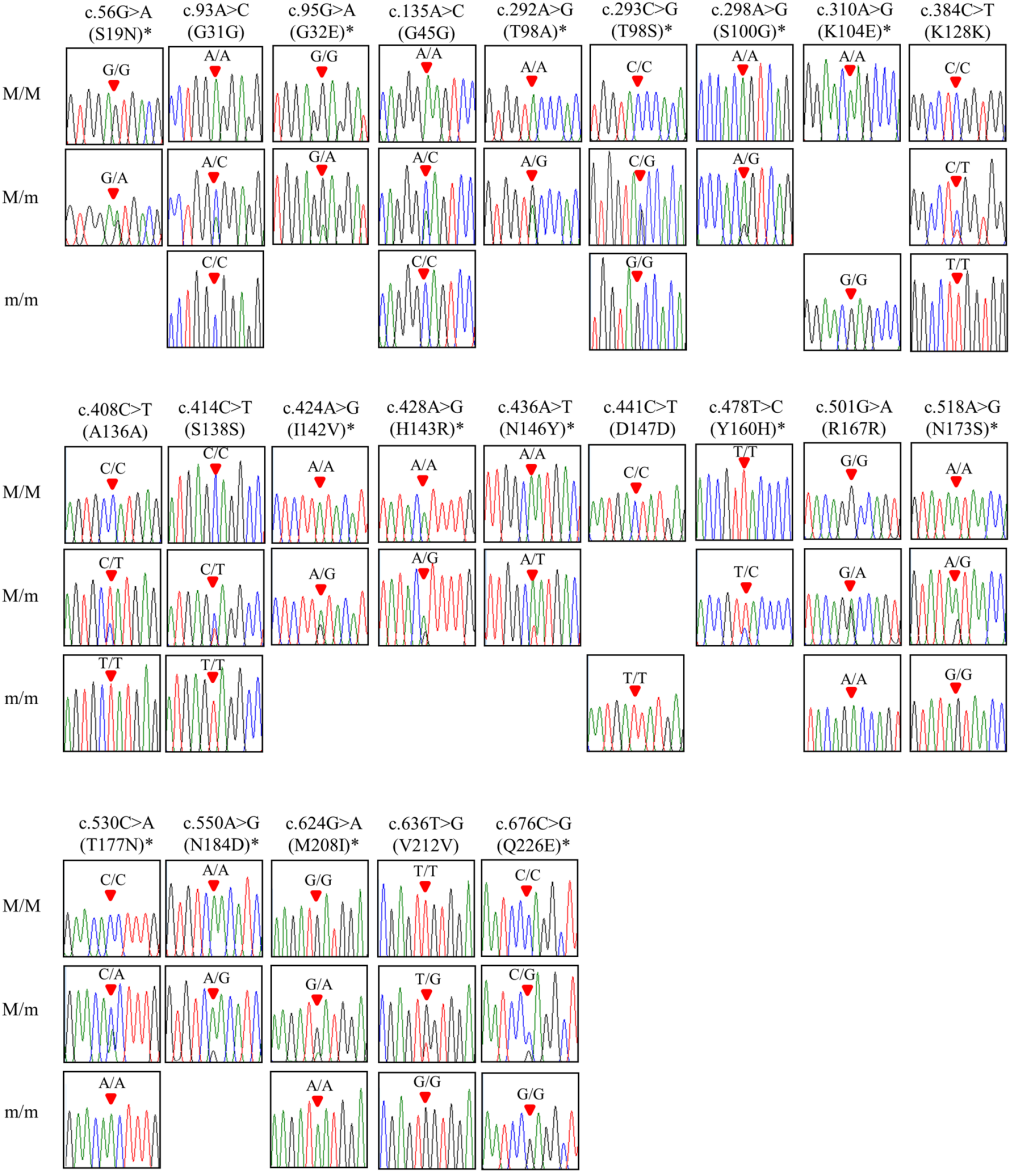
**Electropherograms of 23 novel SNP of the *PRNP* gene found in 248 elk.** The colors of the peaks designate each base of the DNA sequence (green: adenine; red: thymine; blue: cytosine; black: guanine). The red arrows designate the location of the SNP found in the present study. *indicates non-synonymous SNP. M/M: major homozygote; M/m: heterozygote; m/m: minor homozygote.

To identify polymorphisms of the elk* PRNP* gene, we performed amplicon sequencing analysis targeting the ORF of the elk* PRNP* gene. We identified a total of 26 SNP, including 10 synonymous SNP and 16 non-synonymous SNP. Among 26 SNP, 23 SNP were novel SNP, including 8 synonymous SNP and 15 non-synonymous SNP (Figures 1 and 2). We also found c.63C > T, G (V21V), c.312G > A (K104K) and c.394A > T (M132L) SNP reported in elk in previous studies [12].

### Identification of a strong association between* PRNP* SNP and susceptibility to CWD in elk

To investigate the relationship of *PRNP* SNP with susceptibility to CWD, we compared the genotype, allele and haplotype distributions between 196 CWD-negative and 52 CWD-positive elk. Detailed information on the genotype, allele and haplotype distributions is described in Tables 1 and 2. Notably, the genotype and allele distributions of the c.63C > T, G (V21V), c.298A > G (S100G) and c.408C > T (A136A) SNP were significantly different between CWD-negative and CWD-positive elk. In addition, allele distributions of c.312G > A (K104K), c.384C > T (L128L) and c.501G > A (R167R) were significantly different between CWD-negative and CWD-positive elk. As shown in a previous study, we did not find an association of c.394A > T (M132L SNP) with susceptibility to CWD in elk [12].

**Table 1:** **Comparison of genotype and allele distributions of the prion protein gene** (*PRNP*)** between chronic wasting disease (CWD)-negative and CWD-positive elks**

Polymorphisms			Genotype frequencies, n			Allele frequencies, n		HWE	*P*-value^a^	*P*-value^b^
			MM	Mm	mm	M	m			
c.56G > A	S19N	CWD-neg	188	8	0	384	8	0.7705	1	1
		CWD-pos	50	2	0	102	2	0.8875		
c.63C > T, G	V21V	CWD-neg	88	84	24	260	132	0.5701	**0.0322**	**0.0024**
		CWD-pos	34	17	1	85	19	0.4945		
c.93A > C	G31G	CWD-neg	192	3	1	387	5	0	1	0.589
		CWD-pos	52	0	0	104	0	N.A		
c.95G > A	G32E	CWD-neg	195	1	0	391	1	0.9714	1	1
		CWD-pos	52	0	0	104	0	N.A		
c.135A > C	G45G	CWD-neg	191	4	1	386	6	0	0.6696	0.3516
		CWD-pos	52	0	0	104	0	N.A		
c.292A > G	T98A	CWD-neg	177	19	0	373	19	0.4757	0.0851	0.0918
		CWD-pos	51	1	0	103	1	0.9441		
c.293C > G	T98S	CWD-neg	192	3	1	387	5	0	1	0.589
		CWD-pos	52	0	0	104	0	N.A		
c.298A > G	S100G	CWD-neg	196	0	0	392	0	N.A	** < 0.0001**	** < 0.0001**
		CWD-pos	46	6	0	98	6	0.6588		
c.310A > G	K104E	CWD-neg	195	0	1	390	2	0	1	1
		CWD-pos	52	0	0	104	0	N.A		
c.312G > A	K104K	CWD-neg	139	53	4	331	61	0.685	0.0748	**0.0199**
		CWD-pos	45	7	0	97	7	0.6027		
c.384C > T	L128L	CWD-neg	183	12	1	378	14	0.1198	0.1585	**0.0491**
		CWD-pos	52	0	0	104	0	N.A		
c.394A > T	M132L	CWD-neg	135	50	11	320	72	0.0365	0.8542	0.479
		CWD-pos	38	12	2	88	16	0.4125		
c.408C > T	A136A	CWD-neg	173	22	1	368	24	0.7416	**0.0058**	**0.0052**
		CWD-pos	37	15	0	89	15	0.2242		
c.414C > T	S138S	CWD-neg	188	7	1	383	9	0.0043	0.4868	0.215
		CWD-pos	52	0	0	104	0	N.A		
c.424A > G	I142V	CWD-neg	195	1	0	391	1	0.9714	1	1
		CWD-pos	52	0	0	104	0	N.A		
c.428A > G	H143R	CWD-neg	193	3	0	389	3	0.914	1	1
		CWD-pos	51	1	0	103	1	0.9441		
c.436A > T	N146Y	CWD-neg	195	1	0	391	1	0.9714	1	1
		CWD-pos	52	0	0	104	0	N.A		
c.441C > T	D147D	CWD-neg	195	0	1	390	2	0	1	1
		CWD-pos	52	0	0	104	0	N.A		
c.478 T > C	Y160H	CWD-neg	195	1	0	391	1	0.9714	1	1
		CWD-pos	52	0	0	104	0	N.A		
c.501G > A	R167R	CWD-neg	183	12	1	378	14	0.1198	0.1585	**0.0491**
		CWD-pos	52	0	0	104	0	N.A		
c.518A > G	N173S	CWD-neg	192	3	1	387	5	0	1	0.589
		CWD-pos	52	0	0	104	0	N.A		
c.530C > A	T177N	CWD-neg	186	9	1	381	11	0.2677	0.3762	0.1312
		CWD-pos	52	0	0	104	0	N.A		
c.550A > G	N184D	CWD-neg	195	1	0	391	1	0.9714	1	1
		CWD-pos	52	0	0	104	0	N.A		
c.624G > A	M208I	CWD-neg	190	4	2	384	8	0	0.7389	0.2139
		CWD-pos	52	0	0	104	0	N.A		
c.636 T > G	V212V	CWD-neg	187	7	2	381	11	0	0.4741	0.1312
		CWD-pos	52	0	0	104	0	N.A		
c.676C > G	Q226E	CWD-neg	165	28	3	358	34	0.1689	0.5108	0.7639
		CWD-pos	42	10	0	94	10	0.4429		

Bold texts indicate* P* < 0.05.

^a^compared genotype distributions between CWD-negative and CWD-positive elks.

^b^compared allele distributions between CWD-negative and CWD-positive elks.

CWD-neg: CWD-negative elks, CWD-pos: CWD-positive elks, HWE: Hardy–Weinberg equilibrium, M: major allele, m: minor allele, MM: major homozygote, Mm: heterozygote, mm: minor homozygote, N.A: not applicable.

**Table 2:** **Comparison of haplotype distributions of the prion protein gene** (*PRNP)*** between chronic wasting disease (CWD)-negative and CWD-positive elks**

Haplotype	c.56G > A	c.95G > A	c.292A > G	c.293C > G	c.298A > G	c.310A > G	c.394A > T	c.424A > G	c.428A > G	c.436A > T	c.478 T > C	c.518A > G	c.530C > A	c.550A > G	c.624G > A	c.676C > G	CWD Negative (*n* = 392)	CWD Positive (*n* = 104)	*P*-value
Ht1	G	G	A	C	A	A	A	A	A	A	T	A	C	A	G	G	280 (0.715)	73 (0.704)	0.8046
Ht2	G	G	A	C	A	A	T	A	A	A	T	A	C	A	G	G	63 (0.161)	14 (0.130)	0.3542
Ht3	G	G	G	C	A	A	A	A	A	A	T	A	C	A	G	C	17 (0.042)	0 (0)	**0.0300**
Ht4	A	G	A	C	A	A	A	A	A	A	T	A	C	A	G	C	6 (0.015)	2 (0.019)	0.6760
Ht5	G	G	A	C	A	A	A	A	A	A	T	A	C	A	G	C	4 (0.011)	7 (0.070)	**0.0023**
Others																	22 (0.056)	8 (0.077)	

Bold texts indicate statistical significance (*P* < 0.05).

The most frequently observed haplotype was GGACAAAAAATACAGG (CWD-negative elk: 71.5%; CWD-positive elk: 70.4%), followed by GGACAATAAATACAGG (CWD-negative elk: 16.1%; CWD-positive elk: 13%) and GGGCAAAAAATACAGC (CWD-negative elk: 4.2%; CWD-positive elk: 0%). Notably, the GGGCAAAAAATACAGC and GGACAAAAAATACAGC haplotype distributions were significantly different between CWD-negative and CWD-positive elk (Table 2).

We investigated the LD among the 16 non-synonymous SNP of the elk* PRNP* gene with r^2^ values. The detailed LD values are described in Table 3. In the CWD-positive elk, all of the SNP showed a weak LD (r^2^ < 0.333). In the CWD-negative elk, 11 strong LD were found among 16 non-synonymous SNP. LD distributions were significantly different between CWD-negative and CWD-positive elk.

**Table 3:** **Linkage disequilibrium (LD) among non-synonymous single nucleotide polymorphisms (SNP) of the cervid prion protein gene** (*PRNP*)** in elks**

r^2^	c.56G > A	c.95G > A	c.292A > G	c.293C > G	c.298A > G	c.310A > G	c.394A > T	c.424A > G	c.428A > G	c.436A > T	c.478 T > C	c.518A > G	c.530C > A	c.550A > G	c.624G > A	c.676C > G
c.56G > A	−	−	0	−	0.001	−	0.004	−	0	−	−	−	−	−	−	0.002
c.95G > A	0	−	−	−	−	−	−	−	−	−	−	−	−	−	−	−
c.292A > G	0	0	−	−	0.001		.002	−	0	−	−	−	−	−	−	0.091
c.293C > G	0	0	0.001	−	−	−	−	−	−	−	−	−	−	−	−	−
c.298A > G	−	−	−	−	−	−	0.029	−	0.001	−	−	−	−	−	−	0.001
c.310A > G	0	0	0	0	−	−	−	−	−	−	−	−	−	−	−	−
c.394A > T	0.003	0.011	0.011	0.003	−	0.023	−		0.002	−	−	−	−	−	−	0.019
c.424A > G	0	0	0	0	−	0	0.011	−	−	−	−	−	−	−	−	−
c.428A > G	0	0	0	**0.597**	−	0	0.002	0	−	−	−	−	−	−	−	0.091
c.436A > T	0	0	0	0	−	0	0.001	0	0	−	−	−	−	−	−	−
c.478 T > C	0	0	0	0	−	0	0.011	**1.0**	0	0	−	−	−	−	−	−
c.518A > G	0	0	0.001	**1.0**	−	0	0.003	0	**0.597**	0	0	−	−	−	−	−
c.530C > A	0.001	0	0.001	**0.447**	−	0	0.006	0	0.267	0	0	**0.447**	−	−	−	−
c.550A > G	0	**1.0**	0	0	−	0	0.011	0	0	0	0	0	0	−	−	−
c.624G > A	0	0	0.001	**0.62**	−	0	0.001	0	**0.37**	0	0	**0.62**	**0.547**	0	−	−
c.676C > G	0.002	0	**0.414**	0.136	−	0	0.008	0	0.081	0	0	0.136	0.244	0	0.219	−

The above diagonal indicates the LD value in CWD-positive elks. Below the diagonal indicates the LD value in CWD-negative elks. Bold texts indicate strong LD (r^2^ > 0.333).

### In silico evaluation of the S100G SNP on elk PrP

**Figure 3:**
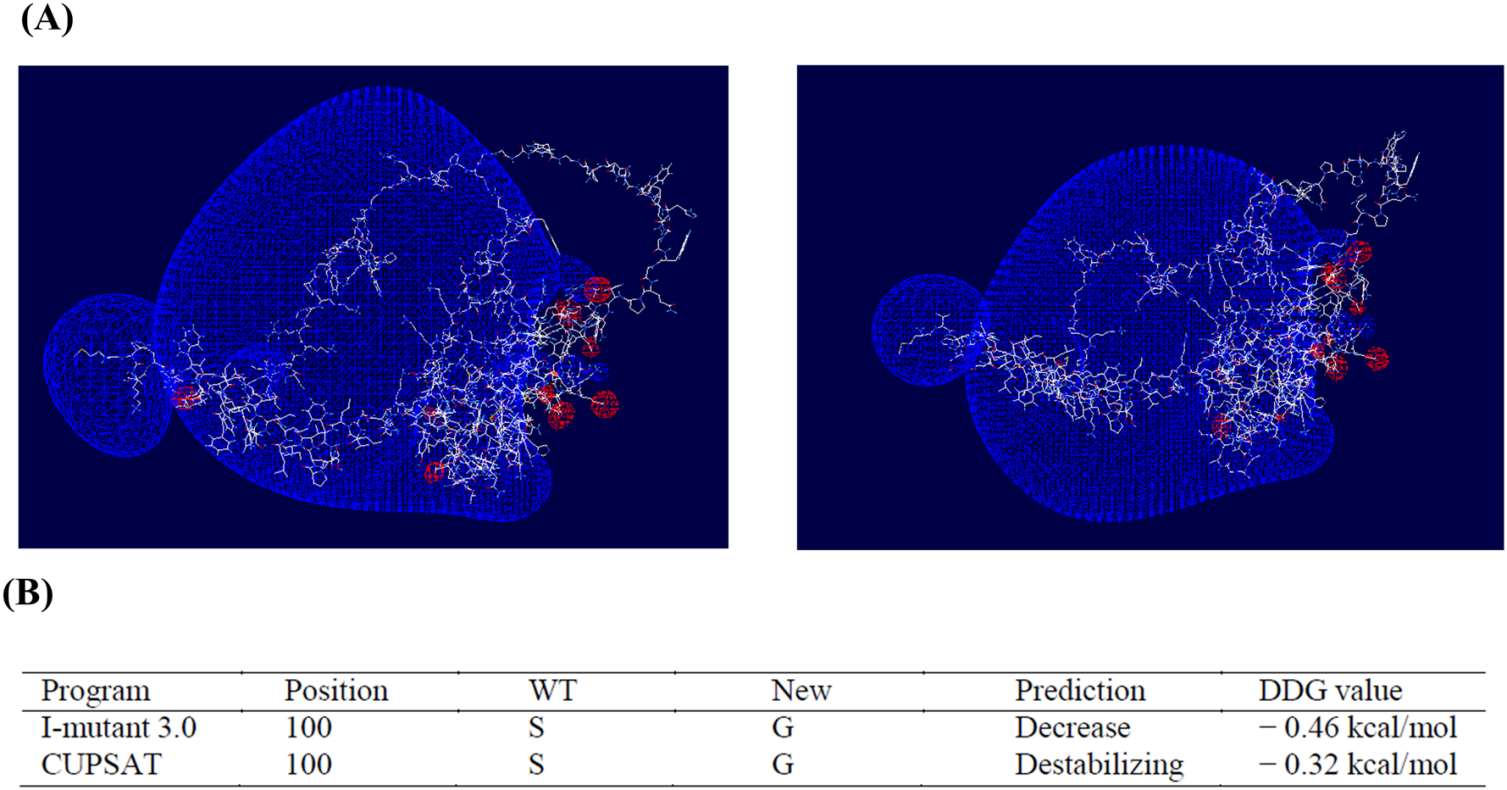
**In silico analyses of elk PrP according to S100G.** **A** Electrostatic potential and 3D structure analysis of elk PrP. The left panel indicates wild-type elk PrP. The right panel indicates elk PrP with the G100 allele. Positive potentials are noted in blue. Negative potentials are drawn in red. **B** Prediction of protein stability changes using I-mutant 3.0 and CUPSAT. The DDG value indicates the free energy change according to S100G.

First, the 3D structures of wild-type (S100) and mutant (G100) elk PrP were predicted by AlphaFold. Then, the predicted structure was visualized with Swiss-PdbViewer, and the electrostatic potential was analyzed (Figure 3A). Notably, the positive potential of elk PrP with the G100 allele was shrank compared to that of wild-type elk PrP.

We estimated the protein stability changes according to S100G by I-mutant 3.0 and CUPSAT (Figure 3B). Notably, S100G was predicted to induce a decrease in the free energy of elk PrP (I-mutant 3.0: − 0.46 kcal/mol; CUPSAT: − 0.32 kcal/mol).

## Discussion

In the present study, we found 23 novel SNP of the elk* PRNP* gene and a high level of genetic diversity (Table 1, Figures 1, 2). However, a previous study using microsatellite analysis has reported that elk have low genetic diversity [24]. Previous studies have reported that genetic diversity of the* PRNP* gene is correlated to prion resistance and susceptibility. A small number of SNP have been reported in dogs and horses, which are prion-resistant animals [25–27]. In contrast, sheep, goats, cattle, deer, and humans, which are prion-susceptible animals, show high genetic diversity for the* PRNP* gene [8, 28–31]. This phenomenon may provide clues to explain the high genetic diversity of the elk* PRNP* gene. Moreover, given that this population was originally imported from Canada, it is possible that the observed phenomenon is a result of its unique history, specific management practices, or animal relocation. Therefore, further investigation of this issue would be highly valuable in the future.

We also identified a strong association between *PRNP* polymorphisms and susceptibility to CWD in elk (Tables 1 and 2). Among those SNP, the S100G SNP is the only non-synonymous SNP. In addition, c.298A > G (S100G) did not have strong LD in CWD-positive and CWD-negative elk (Table 3). Since the non-synonymous SNP directly affects the structural features of the protein, we generated the template of elk PrP according to the S100G SNP by AlphaFold and analyzed the 3D structure and electrostatic potential (Figure 3A). Although the 3D structure of wild-type elk PrP was not significantly different from that of elk PrP with the G100 allele, notably, the positive charge of elk PrP with the G100 allele was decreased compared to that of wild-type elk PrP. In addition, the free energy of elk PrP with the G100 allele was decreased compared to that of wild-type PrP (Figure 3B). Previous studies have reported that the electrostatic potential of PrP plays an important role in PrP oligomerization [32]. In addition, a large free energy barrier is a crucial factor affecting protein stability, and unstable PrP is related to amyloid propensity [33, 34]. Thus, the S100G SNP was predicted to alter the electrostatic structure of elk PrP and provide a susceptible feature to CWD. Further validation using prion infection in transgenic mice and protein misfolding cyclic amplification (PMCA) and RT-QuIC assays with elk PrP carrying S100G is needed to evaluate the relationship between the S100G SNP and susceptibility to CWD in the future.

CWD is the most potent infectious property among prion diseases [35]. CWD is regarded to be transmitted through direct animal contact or by indirect exposure to contaminated environmental factors [36]. In addition, recent studies have reported that sporadic forms of CWD have emerged in Northern European countries [35, 37]. Furthermore, several cases of transmission by overcoming the interspecies barrier have been reported, and experimental infection of CWD agents caused CWD-related phenotypes in nonhuman primates [38]. In Korea, meat and antlers derived from Cervidae species are frequently consumed for food or oriental medicine. Thus, careful preemptive control of CWD is needed. For the preemptive control of CWD in elk, culling for individuals with CWD-related genotypes is also a good method, and the S100G SNP presented in this study is also proposed as a potential candidate for the construction of a selective breeding system. Since *PRNP* polymorphisms are related to not only susceptibility to CWD but also modulation of strain selection [39, 40], it is highly desirable to investigate the characteristics of S100G SNP as a novel CWD strain to construct the selective breeding system in the future.

In conclusion, we found 23 novel SNP of the elk* PRNP* gene. We identified a strong association between* PRNP* SNP and susceptibility to CWD in elk. S100G SNP is predicted to decrease the electrostatic potential and free energy of elk PrP. To the best of our knowledge, this is the first report of a strong association between the S100G SNP and susceptibility to CWD.

## Data Availability

All data are available from the corresponding authors upon reasonable request.
